# In Vitro Gastrointestinal Bioaccessibility of the Phenolic Fraction from *Agave inaequidens* Flower

**DOI:** 10.3390/foods14132375

**Published:** 2025-07-04

**Authors:** Imelda N. Monroy-García, Laura Lucely González-Galván, Catalina Leos-Rivas, Mayra Z. Treviño-Garza, Eduardo Sánchez-García, Ezequiel Viveros-Valdez

**Affiliations:** 1 Departamento de Ingeniería Química y Bioquímica, Tecnológico Nacional de Mexico, Instituto Tecnológico de Los Mochis, Juan de Dios Bátiz y 20 de Noviembre, Los Mochis 81259, Sinaloa, Mexico; imelda.mg@mochis.tecnm.mx; 2 Facultad de Ciencias Biológicas, Universidad Autónoma de Nuevo León, Av. Pedro de Alba S/N, San Nicolás de los Garza 66450, Nuevo León, Mexico; lucely.gonzalezg@uanl.edu.mx (L.L.G.-G.); catalina.leosrs@uanl.edu.mx (C.L.-R.); mayra.trevinogrz@uanl.edu.mx (M.Z.T.-G.); eduardo.sanchezgrc@uanl.edu.mx (E.S.-G.)

**Keywords:** edible flowers, biological activity, enzyme inhibition, in vitro digestion

## Abstract

Edible flowers are gaining recognition as rich sources of nutrients and phytochemicals. In Mexico, the flower of *Agave inaequidens* has been traditionally consumed since pre-Hispanic times. This study investigated its nutritional profile and the in vitro gastrointestinal bioaccessibility of its phenolic fraction. During in vitro digestion (oral, gastric, and intestinal), the total phenolic content of *A. inaequidens* significantly decreased from 138 to 21 mg GAE/100 g DW (15.22% bioaccessibility), while total flavonoid content dropped from 8 to 4.6 mg CE/100 g DW (57.5% bioaccessibility). Consequently, antioxidant activity, assessed by ABTS, DPPH, and hemolysis inhibition assays, also declined post-digestion. Interestingly, the digestive process modulated the flower’s inhibitory activity against digestive enzymes before and after in vitro digestion: α-amylase inhibition slightly decreased (IC_50_ 1.8 to 2.1 mg/mL), but α-glucosidase (IC_50_ 2.7 to 1.6 mg/mL) and lipase (IC_50_ > 3 to 1.4 mg/mL) inhibition increased. The *A. inaequidens* flower is a good source of fiber and low in fat. These findings underscore its potential as a functional food ingredient, offering bioaccessible phenolic compounds with antioxidant and enzyme inhibitory properties.

## 1. Introduction

Bioprospecting, defined as the systematic exploration of natural resources for valuable compounds, is crucial for discovering novel applications of plant-based materials across various industries, including food, cosmetics, pharmaceuticals, and agriculture [1]. Within the food sector, bioprospecting aids in identifying bioactive ingredients and developing functional foods that offer health benefits beyond basic nutrition through biologically active compounds, potentially reducing the risk of chronic diseases [2]. In relation to the above, edible flowers are increasingly recognized as integral components of such foods [3].

The global interest in edible flowers, or floriphagy, is expanding, notably in Mexico, due to their decorative, nutritional, and functional attributes [4]. Scientific research underscores their health-promoting potential, attributing it to essential vitamins, minerals, proteins, amino acids, and a wide range of bioactive compounds [5]. Among these, phenolic compounds are especially noted for their strong antioxidant activity [6].

The agave flower, known by regional names like flor de maguey, bayusas, gualumbos, and cacayas, is a traditional Mexican delicacy, deeply embedded in the culinary practices of several states [7]. Edible agave species include *A. salmiana*, *A. mapisaga*, *A. marmorata*, *A. durangensis*, *A. inaequidens*, *A. americana*, *A. applanata*, *A. lechuguilla*, and *A. striata*, among others [7]. Despite its traditional use, comprehensive research on the composition and biological activity of these flowers remains limited. Previous studies have analyzed the nutritional content of *A. salmiana*, revealing high levels of protein, fiber, and minerals [8]. Furthermore, *A. durangensis* and *A. americana* flowers have been identified as excellent sources of antioxidants [9,10]. However, detailed information regarding the specific functional properties of the agave flower is largely unexplored, necessitating further in-depth investigation.

It is well-known that flowers contain high amounts of phenols and flavonoids, two key groups of plant secondary metabolites known for their significant biological activities. In edible flowers, these compounds exhibit anti-inflammatory, anticancer, and antidiabetic properties [11]. The Agave genus is particularly rich in phenolic compounds, including flavonoids, homoisoflavonoids, and phenolic acids, which are associated with diverse biological activities such as antioxidant, antibacterial, antifungal, antinematode, and immunomodulatory effects [12]. Agave phenols also serve as valuable chemotaxonomic markers for species classification [12].

Furthermore, certain flavonoid-rich flowers have demonstrated the ability to inhibit key digestive enzymes, such as α-amylase and α-glucosidase, which is significant for glycemic control and potential antidiabetic effects [13]. Inhibition of lipase, another crucial digestive enzyme modulated by phenolic compounds, is linked to a reduced risk of cardiovascular diseases, hypertension, and obesity [13]. Therefore, it is hypothesized that agave flower extract will also exhibit inhibitory activity against lipase.

Since the efficacy of bioactive compounds depends on their bioaccessibility (their release from the food matrix during digestion and subsequent absorption) [14], this study evaluates the biological effects of enriched phenolic fraction from the agave flower before and after simulated digestion. This approach enables a more accurate assessment of its health potential. The research aims to uncover new insights into the antioxidant and enzyme-inhibitory properties of *Agave inaequidens* flowers, supporting their valorization as a functional food ingredient.

## 2. Materials and Methods

### 2.1. Plant Material Collection and Preparation

Agave flowers were collected in the locality of San Dimas (Durango, Mexico) during their flowering period (April–May). Flowers were harvested from three different agave plants of the same species at an intermediate stage of maturity, when the flowers were still closed and exhibited a greenish-yellow coloration. The taxonomic identification of the plant material was performed by Dr. Martha González Elizondo, Head of the CIIDIR Durango Herbarium, Durango, Mexico, (Voucher Number: 55902). Subsequently, a cleaning process was performed, which involved removing only the immature inflorescence (buds) and discarding the receptacle, peduncle, and branching. The cleaned flowers were maintained under frozen conditions until analysis. Given that agave flowers are traditionally consumed cooked, they were pre-cooked in water at 70 °C for 30 min under atmospheric conditions, prior to analysis.

### 2.2. Proximate Analysis

A proximate analysis was performed to identify the following parameters: moisture (NOM-116-SSA-1994), crude protein (NMX-F-608-NORMEX-2011), ether extract (NOM-086-SSA1-1994), fiber (NMX-F-613-NORMEX-2017), ash (NMX-F-607-NORMEX-2020), and nitrogen-free extract. The moisture and total solids content of the flower samples was determined by drying at 110 °C in an oven until a constant weight was reached (Thermo Scientific Heratherm, Waltham, MA, USA). Crude protein was quantified using the macro-Kjeldahl method, applying a nitrogen-to-protein conversion factor of 6.25. Total fat was extracted via a Soxhlet procedure. Ash content was measured following incineration of the samples at 525 °C for 24 h in a muffle furnace (Nabertherm L 3/11, Lilienthal, Germany). Total carbohydrates were calculated by subtracting the percentages of moisture, crude protein, total fat, and ash from 100%. Finally, the total energy content was estimated using Atwater conversion factors. Results were expressed as a percentage on a dry basis (DB) and wet basis (WB).

### 2.3. Extraction and Fractionation

The extraction method was based on the methodology described by Jimenez-Aspee et al., 2015 [15] with minor modifications. Briefly, pre-cooked flowers were comminuted to reduce particle size. This was followed by three sequential extractions using acidified methanol (MeOH) 0.01% at a 1:3 (*w*/*v*) ratio of plant material to solvent. Each extraction step included sonication for 15 min. The three extracts were then combined, and the solvent was removed under reduced pressure (rotary evaporation) at 37 °C to yield the first concentrated MeOH extract. A portion of this concentrated MeOH extract was subsequently adsorbed onto pre-conditioned Amberlite XAD-7-HP resin. The concentrated extract was dissolved in distilled water and thoroughly mixed with the Amberlite resin at a 1:5 (extract to Amberlite) ratio, with constant agitation for 40 min. The extract-resin mixture was then filtered and washed with water to eliminate undesired components, such as proteins, sugars, and lipids. Finally, MeOH was added to desorb the phenolic compounds from the resin. After recovering the phenolic compounds with MeOH, the solvent was evaporated under reduced pressure at 37 °C to obtain the second phenolic extract (PE). Finally, a fractionation step with ethyl acetate was performed. A portion of the PE was dissolved in distilled water, followed by liquid-liquid extraction using ethyl acetate at a 1:2 (*v*/*v*) ratio. The separated ethyl acetate fraction was dried over sodium sulfate and filtered, and the solvent was evaporated by simple distillation under reduced pressure at 37 °C to yield the third enriched phenolic extract (EPE).

The extraction yield for all four obtained extracts was calculated using the following formula:

Yield (%) = (Weight of extract/Weight of sample) × 100

### 2.4. In Vitro Gastrointestinal Digestion

The bioaccessibility of phenolic compounds during in vitro gastrointestinal digestion is hypothesized to be influenced by a complex interplay of factors, including food matrix interactions, chemical structure of phenolics (e.g., glycosylation, polymerization, lipophilicity), and digestive conditions (changes in pH, enzymes, bile salts). These factors significantly impact the stability, solubility, and potential degradation or transformation of phenolic compounds [16].

A portion of the EPE extract was subjected to in vitro gastrointestinal digestion, employing a three-phase sequential digestion system: oral, gastric, and intestinal. This methodology was based on the approach described by Morais et al., 2020 [16] with some modifications. A fraction of the EPE extract was vigorously mixed and dissolved in distilled water. For the oral phase, 25 mL of the sample were mixed with 5 mL of simulated oral fluid (2.38 g of Na_2_HPO_4_, 0.19 g of KH_2_PO_4_, 8 g of NaCl, and 200 U/L α-amylase dissolved in one liter of distilled water). This mixture was incubated under light protection with constant agitation at 90 rpm for 10 min at 37 ± 2 °C and pH = 7. The gastric phase was conducted by adding 1 mL of simulated gastric fluid (0.108 g of pepsin dissolved in 10 mL of 0.1 M HCl), and the pH was adjusted to 2.0 with 6 M HCl. The mixture was then re-incubated under the same conditions for 2 h. Finally, for the intestinal phase, 2.5 mL of the pancreatin solution (0.08 g of pancreatin dissolved in 10 mL of 0.5 M NaHCO_3_) and 2.5 mL of the bile salts solution (0.5 g of bile salts were dissolved in 10 mL of 0.5 M NaHCO_3_) were added. The pH was adjusted to 7 with 6 M NaOH. The mixture was then re-incubated under the same conditions for 2 h.

Upon completion of the digestion, a liquid-liquid extraction with ethyl acetate (100 mL × 3 times) was performed on the digested material. The phenolic compounds present in the ethyl acetate phase were concentrated by simple distillation under reduced pressure at 37 °C to obtain the fourth extract, the digested enriched phenolic extract (DEPE).

To determine the bioaccessibility of the bioactive compounds from the *A. inaequidens* flower, the following equation was used:

Bioaccessibility (%) = (Digested Compounds/Undigested Compounds) × 100

### 2.5. Total Phenolic Content

Total phenolic content was determined using the Folin–Ciocalteu (FC) method. This method relies on the transfer of electrons from phenolic groups to phosphomolybdic and phosphotungstic acids in an alkaline environment. Extracts (MeOH, PE, EPE, DEPE) were initially dissolved in distilled water (10 mg/mL) [17]. In triplicate, 100 μL of each extract were combined with 250 μL of 1N FC reagent, 1250 μL of 20% (*w*/*v*) Na_2_CO_3_, and 400 μL of distilled water. The mixtures were incubated for 2 h at room temperature in darkness. Subsequently, 200 μL of each reaction mixture were transferred to a 96-well microplate, and absorbance was measured at 760 nm using a microplate spectrophotometer (Agilent BioTek Epoch Microplate Spectrophotometer, Winooski, VT, USA). Gallic acid (10–1000 μg/mL) was used as a standard (y = 10.329x + 0.1454; R^2^ = 0.9952), and results were expressed as mg of gallic acid equivalents (GAE) per 100 g of fresh weight.

### 2.6. Total Flavonoid Content

Total flavonoid content was quantified using the colorimetric aluminum chloride (AlCl_3_) method, which involves the formation of stable aluminum-flavonoid complexes in a basic medium. The procedure was performed based on Monroy-García et al., 2024 [17]. Extracts (MeOH, PE, EPE, DEPE) were dissolved in MeOH (10 mg/mL). Subsequently, 200 μL of each extract were mixed with 1250 μL of distilled water and 75 μL of 5% (*w*/*v*) NaNO_2_. The mixture was incubated for 5 min at room temperature, protected from light. Then, 150 μL of 10% (*w*/*v*) AlCl_3_ were added, followed by a one min incubation. Finally, 500 μL of 1M NaOH and 325 μL of distilled water were added. Two hundred μL of the final solution were transferred to a 96-well microplate, and absorbance was measured at 510 nm using a microplate spectrophotometer (Agilent BioTek Epoch Microplate Spectrophotometer). Catechin (50–500 μg/mL) served as the standard (y = 0.0124x + 0.0173; R^2^ = 0.9995), and results were expressed as mg of catechin equivalents (CE) per 100 g of fresh weight.

### 2.7. Antioxidant Activity

#### 2.7.1. DPPH• Radical Scavenging Activity Assay

The 2,2-diphenyl-1-picrylhydrazyl (DPPH•) assay is a colorimetric method used to evaluate the free radical scavenging potential of antioxidant molecules. The procedure was conducted following the method proposed by Monroy-García et al., 2024 [17]. Extracts (MeOH, PE, EPE, DEPE) were initially dissolved in MeOH (1 mg/mL). Then, 100 μL of each serially diluted extract were added to wells of a 96-well microplate, followed by the addition of 100 μL of 150 μM DPPH• radical solution (80% MeOH). The mixtures were incubated for 30 min at room temperature, protected from light. Finally, absorbance was measured at 517 nm using a microplate spectrophotometer (Agilent BioTek Epoch Microplate Spectrophotometer). The absorbance values were converted to percentage of anti-radical activity (AA%) using the following formula:

AA% = 100 − [(Am − Ac)/Ac] × 100

where Ac and Am represent the absorbance of the control and the sample, respectively. Trolox was used as a positive control, and MeOH served as a negative control. The assay was performed in triplicate, and radical scavenging activity was expressed as half the maximal effective concentration (EC_50_) in mg/mL.

#### 2.7.2. ABTS•+ Radical Scavenging Ability Assay

The ABTS•+ radical scavenging ability assay is a spectrophotometric technique that assesses antioxidant capacity based on the inhibition of the 2,2′-azino-bis-3-ethylbenzothiazoline-6-sulfonic acid radical cation (ABTS•+). The procedure followed modified methods by Monroy-García et al., 2021 [17]. The ABTS•+ radical was prepared by mixing 7 mM ABTS in an aqueous solution with 2.45 mM potassium persulfate (K_2_S_2_O_8_), followed by a 16 h incubation at room temperature in the dark. Once formed, the ABTS•+ radical mixture was diluted with distilled water until an absorbance of 0.7 ± 0.02 was achieved at 734 nm using a microplate spectrophotometer. Subsequently, the reactive mixture was prepared in a 96-well microplate by combining 20 μL of each extract (MeOH, PE, EPE, DEPE), dissolved in MeOH (1 mg/mL), with 200 μL of the ABTS•+ radical solution. The mixtures were incubated for 20 min in the dark, and, finally, absorbance was measured at 734 nm (Agilent BioTek Epoch Microplate Spectrophotometer). Trolox was used as a standard, and results were expressed as μM Trolox equivalents per 100 g of fresh weight.

#### 2.7.3. Protective Hemolysis Erythrocyte Induced by the Radical 2,2′-Azobis(2-amidinopropane) Dihydrochloride (AAPH)

This method evaluates the protective effect of compounds against hemolysis induced by peroxyl radicals generated by AAPH. AAPH-induced oxidation of erythrocyte membrane lipids and proteins leads to hemolysis.

To obtain the blood, the following procedure was followed. The research protocol was reviewed and approved by the Department of Chemistry of the FCB/UANL, protocol code ATL-03-2023, approved in May 2023. Special consideration was given to participant privacy and data security. The participants received a comprehensive explanation of the study objectives and procedures and provided voluntary, written informed consent.

Initially, a 10 mL blood sample was collected from a healthy individual via venipuncture into EDTA-containing tubes. The blood sample was centrifuged at 1500 rpm for 10 min to separate red blood cells, white blood cells, and plasma. The red blood cells (erythrocytes) were then separated from the plasma and washed three times with 5 volumes of saline buffer (PBS; 120 mM NaCl, 22.21 mM Na_2_HPO_4_, 5.59 mM KH_2_PO_4_, and 5.5 mM glucose *w*/*v*) at pH 7.4. Each wash was followed by centrifugation at 1500 rpm for 2 min. Subsequently, an erythrocyte suspension was prepared in 4 volumes of PBS solution to achieve a concentration of 8 × 10^9^ cells/mL [18].

The anti-hemolytic activity of the extracts (MeOH, PE, EPE, DEPE) was determined following a methodology adapted from Yang et al., 2017 [19]. Briefly, 250 μL of erythrocyte suspension were mixed with 250 μL of extracts diluted in PBS at various concentrations (250, 500, 1000, and 2000 μg/mL). Finally, 250 μL of 300 mM AAPH in PBS were added, and the mixture was gently shaken for 3 h at 37 °C. After incubation, the mixture was diluted to 8 volumes in PBS and centrifuged at 4000 rpm for 5 min. The absorbance of the supernatant was measured at 540 nm using a microplate spectrophotometer (Agilent BioTek Epoch Microplate Spectrophotometer). The percentage of inhibition was calculated using the following equation:

% of inhibition = [AAAPH − Am]/[AAAPH] × 100

where AAAPH is the absorbance of AAPH at 540 nm, and Am is the absorbance of the extracts at 540 nm. The half maximal inhibitory concentration (IC_50_) of the extract was calculated from a dose-response curve by plotting the percentage of hemolysis inhibition against the extract concentration. Ascorbic acid was used as a positive control, and plain water served as a negative control. Results were expressed as the IC_50_.

### 2.8. Inhibitory Activity of Digestive Enzymes

#### 2.8.1. α-Amylase Inhibitory Activity

The α-amylase inhibitory assay was conducted to assess the capacity of the extracts (MeOH, PE, EPE, DEPE) to inhibit starch hydrolysis by the α-amylase enzyme. The method was adapted from Burgos et al., 2017 [20]. Briefly, 50 μL of serially diluted extracts (5 mg/mL in PBS, pH 6.8) were added to a 96-well microplate, followed by 50 μL of α-amylase solution (1 U/mL in PBS). The mixture was incubated at 37 °C for 15 min. Subsequently, 50 μL of 0.5% (*w*/*v*) starch in PBS were added, and incubation continued for another 20 min at 37 °C. The reaction was terminated by adding 20 μL of 1M HCl, and, finally, 50 μL of iodine reagent (3 mM I_2_ and 30 mM KI) were added. Absorbance was measured at 750 nm using a microplate spectrophotometer (Agilent BioTek Epoch Microplate Spectrophotometer). The percentage of inhibition was calculated using the following formula:

% of activity inhibition = [(Ac − Am)/Ac] × 100

where Ac and Am represent the absorbance of the control and the sample, respectively. Acarbose was used as a positive control, and PBS served as a negative control. Results were expressed as the half maximal inhibitory concentration (IC_50_), calculated using logistic regression analysis.

#### 2.8.2. α-Glucosidase Inhibitory Activity

The α-glucosidase inhibitory assay evaluated the ability of the extracts (MeOH, PE, EPE, DEPE) to inhibit the enzymatic hydrolysis of a synthetic substrate, *p*-nitrophenyl-α-D-glucopyranoside (*p*-NPG), by α-glucosidase. Following procedures by Monroy-García et al., 50 μL of serially diluted extracts (5 mg/mL in PBS, pH 6.8) were placed in a 96-well microplate [18]. Then, 50 μL of α-glucosidase solution (0.8 U/mL in PBS) were added, and the mixture was incubated at 37 °C for 15 min. Subsequently, 50 μL of 625 mM *p*-NPG substrate were added, followed by another 15 min incubation at 37 °C. The reaction was stopped by adding 100 μL of 0.2 M Na_2_CO_3_. Absorbance was measured at 405 nm using a microplate spectrophotometer (Agilent BioTek Epoch Microplate Spectrophotometer). The percentage of inhibition was calculated using the following formula:

% of activity inhibition = [(Ac − Am)/Ac] × 100

where Ac and Am represent the absorbance of the control and the sample, respectively. Acarbose was used as a positive control, and PBS served as a negative control. Results were expressed as the half maximal inhibitory concentration (IC_50_), determined via logistic regression analysis.

#### 2.8.3. Pancreatic Lipase Inhibitory Activity

The inhibitory activity against pancreatic lipase was assessed using *p*-nitrophenol palmitate (*p*-NPP) as a substrate. For this analysis, pancreatic lipase was dissolved (20 mg/mL) in 60 mM PBS (pH 8) and centrifuged at 3500 rpm for 10 min at 4 °C to collect the supernatant for the assay. Following the modified method by Monroy-García et al., 100 μL of serially diluted extracts (5 mg/mL in PBS) were mixed with 30 μL of the lipase solution in a 96-well microplate and incubated for 15 min at 37 °C [18]. After incubation, 10 μL of 10 mM *p*-NPP (in DMSO) were added, and the mixture was re-incubated at 37 °C for 30 min. Absorbance was measured at 405 nm using a microplate spectrophotometer (Agilent BioTek Epoch Microplate Spectrophotometer). The percentage of inhibition was calculated using the following formula:

% of activity inhibition = [(Ac − Am)/Ac] × 100

where Ac and Am represent the absorbance of the control and the sample, respectively. Orlistat was used as a positive control, and PBS served as a negative control. Results were expressed as the half maximal inhibitory concentration (IC_50_), calculated through logistic regression analysis.

### 2.9. Statistical Analysis

All values were expressed as the mean ± standard deviation (*n* = 3). Differences between means were determined using factorial analysis of variance (ANOVA), followed by mean comparison using Student’s *t*-test. Statistical analysis was performed using OpenStat statistical software (version 11.9.08). Statistical significance was set at *p* < 0.05.

## 3. Results and Discussion

The macronutrient content of cooked *A. inaequidens* flowers was determined, as boiling is a common practice for their preparation and consumption [7]. As shown in Table 1, the primary component was water (88.70%), followed by nitrogen-free extract (NFE) at 81.24%, fiber at 7.52%, ash at 6.11%, protein at 3.19%, and ether extract at 1.95%. Compared to raw *Agave salmiana* flowers, which reported 88.10% moisture, 9.65% fiber, 5.65% ash, 11.58% protein, 1.58% ether extract, and 71.58% NFE, the cooked *A. inaequidens* flowers showed no significant nutritional loss despite the cooking process [21]. The most notable differences were observed in protein (11.58% vs. 3.19%) and fiber (9.65% vs. 7.52%).

While cooking can alter nutrient concentration and bioavailability, leading to potential reductions in water-soluble components like proteins and minerals, the nutritional profile of boiled *A. inaequidens* flowers remained comparable to other raw edible flowers, such as *Tropaeolum majus*, *Tagetes erecta*, and *Rosa grandiflora*. These comparisons revealed similar values for ash (0.63–0.80% FW), ether extract (0.23–0.33% FW), and carbohydrates (7.14–14.15% FW), although protein (1.32–1.99% FW) and fiber (3.20–9.20% FW) content varied more significantly [22,23]. Furthermore, the macronutrient composition of boiled *A. inaequidens* flowers was comparable to that of commonly cooked vegetables like cabbage, green pepper, and carrots, which typically contain 0.77–1.27% protein, 0.31–0.59% ash, 0.06–0.20% ether extract, and 5.51–6.70% NFE on a WB [24].

The yield of Agave flower extracts and fractions is presented in Table 2. A decreasing trend in yield was observed with successive fractionation steps from the initial MeOH extract. The solid-liquid extraction with acidified MeOH (0.01%) showed the highest yield at 3.68%. These extracts are known to contain various phytochemicals, including phenolic compounds, carotenoids, coumarins, tannins, alkaloids, terpenoids, steroids, and carbohydrates [25,26]. Subsequent fractionation using Amberlite polymeric resin for phenolic compound adsorption yielded the phenolic extract (PE) with a lower yield of 0.45%. Further partitioning of the PE with ethyl acetate (EtOAc) to isolate more polar phenolic compounds resulted in a significantly reduced yield of 0.15%. Finally, the yield of the ethyl acetate extract after an in vitro digestion (DEPE) process was 0.03%.

Phenolic compounds from plant sources are widely recognized for their diverse health-beneficial effects, including antioxidant, anti-inflammatory, and anticarcinogenic properties. However, the ultimate impact of these compounds on human health is heavily dependent on their bioaccessibility and bioavailability, which are significantly influenced by the complex processes occurring during gastrointestinal digestion [27,28].

During digestion, phenolic compounds undergo various transformations due to changes in pH, enzymatic activity, and interactions with other dietary components. These processes can lead to structural modifications, including hydrolysis, glycosylation, and polymerization. Such alterations can profoundly impact the biological activity of the compounds. For instance, some phenolic compounds may experience a reduction in their biological activity due to degradation or conversion into less active forms [29]. Conversely, digestive processes can sometimes enhance the activity of certain phenolics by releasing them from their complex matrices or transforming them into more bioavailable and potent metabolites [30].

The total phenolic content (TPC) and total flavonoid content (TFC) of *A. inaequidens* flower extracts, both before and after in vitro digestion, are presented in Table 3. Significant variations in TPC and TFC were observed across the extracts. TPC ranged from 16.4 to 46.1 mg GAE/g extract, while TFC ranged from 8.4 to 25.3 mg CE/g extract. Solvent type notably impacted extraction efficiency: the EPE exhibited the highest concentrations of both phenolics (46.1 mg GAE/g) and flavonoids (25.3 mg CE/g), while MeOH extract had the lowest (16.4 mg GAE/g and 8.4 mg CE/g, respectively). Despite the PE’s lower soluble fraction compared to MeOH, its phenolic content was nearly threefold higher.

Evaluation of the PE after in vitro digestion (DPE) revealed a significant decrease in TPC from 46.1 to 7.6 mg GAE/g extract. TFC also decreased from 25.3 to 14.6 mg CE/g extract. Gastrointestinal conditions are known to decrease or transform phenolic compounds, though some may increase [16]. The observed bioaccessibility values for *A. inaequidens* flowers align with reported ranges for other edible flowers, where catechin bioaccessibility varies from 50–172%, quercetin from 1.7–70%, caffeic acid from 4–110%, and syringic acid from 6.6–30% [16]. Similarly, reported bioaccessibility for phenolics and flavonoids in fruit juices is approximately 12.8% and 20.1%, respectively, further supporting our findings for *A. inaequidens* [31].

The TPC of *A. inaequidens* flowers (FW) was 138 mg GAE/100 g, decreasing significantly to 21 mg GAE/100 g after digestion. This fresh weight TPC is comparable to values reported for other edible flowers like sunflower (*Helianthus annuus*), morning glory (*Ipomoea cairica*), iris (*Iris japonica*), lily (*Lilium brownii*), bean flower (*Phaseolus vulgaris*), and sage (*Salvia splendens*), which range from 50 to 156 mg GAE/100 g FW [32]. However, some edible flowers, such as begonia (*Begonia × tuberhybrida*), nasturtium (*Tropaeolum majus*), marigold (*Calendula officinalis*), french marigold (*Tagetes patula*), jasmine (*Jasminum officinale*), and petunia (*Ruellia simplex*), exhibit considerably higher TPC (323–758 mg GAE/100 g FW) [25,33].

The TFC of *A. inaequidens* flowers was 8 mg CE/100 g FW, decreasing to 4.6 mg CE/100 g FW post-digestion. This value is comparable to basil flower (*Ocimum basilicum*) (3.99–21.72 mg CE/100 g FW, depending on region) [34]. In contrast, flowers like begonia (*Begonia boliviensis*), cornflower (*Centaurea cyanus*), rose (*Rosa odorata*), French marigold (*Tagetes patula*), nasturtium (*Tropaeolum majus*), and pansy (*Viola x wittrockiana*) report higher TFCs (135–204 mg rutin equivalents (RE)/100 g FW) [35].

While *A. inaequidens* flowers exhibit similar phenolic concentrations to some edible flowers, significant differences exist compared to others. Notably, the *A. inaequidens* flowers underwent two aggressive processing steps (boiling and in vitro digestion), which can lead to the loss of compounds, particularly water-soluble and thermolabile phenolics. This likely explains the observed lower phenolic content compared to some raw edible flowers and suggests that the *A. inaequidens* results more accurately reflect actual dietary intake. Furthermore, the phenolic content of *A. inaequidens* flowers was found to be higher than that of common cruciferous vegetables like Chinese cabbage (*Brassica rapa* var. chinensis), cabbage (*B. oleracea* var. capitata), cauliflower (*B. oleracea* var. botrytis), broccoli (*B. oleracea* var. italica), and red radish (*Raphanus sativus*), which reported TPC (uncooked) between 35 and 106 mg GAE/100g FW [36].

Antioxidants are crucial for delaying or preventing cellular oxidation. Diets rich in phenolic compounds exhibit significant antioxidant activity, primarily through free radical scavenging, interruption of radical chain reactions, and metal chelation [37]. To assess the antioxidant/anti-radical capacity of edible *A. inaequidens* flowers, three distinct methods were employed: ABTS•+ scavenging ability assay, DPPH• radical scavenging assay, and AAPH-induced hemolysis inhibition.

The results, presented in Table 4, indicate a clear correlation between the concentration of phenolics and flavonoids and antioxidant activity. Higher concentrations of these compounds, achieved through specific extraction methods, consistently led to increased antioxidant efficacy. In the TEAC assay, activity ranged from 19 to 42 μM/mg extract. The phenolic-enriched extract (EPE), with the highest phenolic concentration, demonstrated the strongest activity (42 μM/mg). After in vitro digestion, this activity significantly decreased to 8 μM/mg. Similarly, for the DPPH• radical assay, IC_50_ values ranged from 0.43 to 0.93 mg/mL extract. EPE showed the lowest IC_50_ (0.43 mg/mL), signifying superior activity. Post-digestion, the IC_50_ for EPE increased to 4.2 mg/mL, clearly indicating a reduction in activity. The AAPH-induced hemolysis assay was performed only on EPE and its digested counterpart (DEPE). EPE exhibited an IC_50_ of 70.43 μg/mL. Following in vitro gastrointestinal digestion, the IC_50_ increased to >100 μg/mL, confirming a notable decrease in antioxidant activity, consistent with the other assays.

These findings suggest that the digestion process significantly reduces both the concentration and antioxidant activity of phenolic compounds, aligning with previous research [27,38]. While antioxidant activity may increase during the acidic gastric phase, a considerable decrease often occurs during alkaline intestinal digestion. This is likely due to pH-induced structural changes, including racemization and deprotonation of hydroxyl groups on aromatic rings, which can alter biological responses [39]. Li et al. evaluated the structural stability and antioxidant activity of 27 phenolic compounds (phenolic acids, flavonols, flavonoids, and flavanones) during the in vitro simulated digestion. The results showed lower values of most phenolic compounds after in vitro digestion. Nevertheless, the DPPH, ABTS, and ferric reducing antioxidant power (FRAP) values of phenolic acids and flavonols decreased after in vitro simulated digestion (*p* < 0.05), while the values of DPPH, ABTS, and FRAP of most flavonoids (*p* < 0.05) increased [40]. Similar results were observed on phenolic extracts from olive mill wastewaters. After the digestion process, a massive loss of phenols was found (50−89.3%), coupled with a decrement in antioxidant activity (ABTS•+ and ORAC) [41].

The radical ABTS•+ scavenging ability assay of *A. inaequidens* (5.7 μM/100 g FW) is comparable to that of common vegetables like bitter gourd (7 μM/100 FW) and green cucumber (5 μM/100 FW) [42]. In the DPPH assay, Agave flower showed higher antioxidant activity than three out of four edible flower species analyzed by Pires et al., 2023 (rose: 0.18 mg/mL; dahlia: 0.63 mg/mL; centaurea: 0.83 mg/mL; calendula: 1.37 mg/mL) [43]. For the AAPH-induced hemolysis assay, *A. inaequidens* offered greater protection (IC_50_ of 70.43 μg/mL) before digestion compared to chamomile and pennyroyal (IC_50_ of 127.48 to 129.52 μg/mL) [44]. However, after digestion, the Agave flower’s IC_50_ > 100 μg/mL became similar to these other edible flowers.

Metabolic syndrome, characterized by risk factors like obesity, dyslipidemia, and hyperglycemia, is often managed by inhibiting lipolytic enzymes (lipase) and carbohydrate-metabolizing enzymes (α-amylase and α-glucosidase) [45,46]. While commercial inhibitors like acarbose, voglibose, and orlistat are available, their use is associated with side effects [47]. Consequently, natural inhibitors, particularly phenolic compounds, are being investigated as safer alternatives due to their ability to non-specifically bind and inactivate these enzymes [48]. The inhibitory activity of agave flower extracts, both before and after in vitro digestion, are presented in Table 5. As can be observed, the extraction method influences the phenolic metabolites with enzyme-inhibitory activity. MeOH and PE extracts showed lower activity against the enzymes, while the EPE exhibited notable inhibitory effects.

For α-amylase, EPE showed the highest inhibition (IC_50_ of 1.8 mg/mL), consistent with its higher phenolic content. However, after in vitro gastrointestinal digestion, the IC_50_ increased to 2.1 mg/mL, indicating reduced inhibitory activity. In contrast, α-glucosidase and lipase activities showed an opposite trend post-digestion, with an increase in inhibitory activity. EPE exhibited an α-glucosidase IC_50_ of 2.7 mg/mL, which decreased to 1.6 mg/mL after digestion. Similarly, lipase inhibition by EPE, initially with an IC_50_ > 3 mg/mL, improved to 1.4 mg/mL post-digestion.

This increase in activity is likely attributable to the transformation of phenolic glycosides, prevalent in plant foods, into their corresponding aglycones during digestion [49]. Aglycones, often more bioavailable and potent than their glycoside forms, exhibit stronger bioactivities, including antidiabetic effects [50]. In this regard, the flavonoid profile of the flowers of *A. durangensis* demonstrated the presence of five quercetin glycosides and three kaempferol glycosides; however, several in vitro studies have shown that quercetin and kaempferol aglycones are more potent enzyme inhibitors than their corresponding glycosides [9,51]. Aglycones are generally more lipophilic than their glycosides due to the absence of the polar sugar moiety. This increased lipophilicity facilitates their interaction with the hydrophobic active sites of enzymes, which can lead to stronger binding and, consequently, greater in vitro inhibitory activity [52]. In addition, the presence or absence of sugars at specific positions can influence the availability of hydroxyl groups or other structures crucial for interaction with amino acids in the enzyme’s active site. In many cases, the addition of a glycosyl group can “block” or alter the molecule’s conformation, reducing its ability to fit into the enzyme active site [53]. Thus, in vitro digestion appears to convert phenolic compounds in *A. inaequidens* flowers into more active aglycones, enhancing their α-glucosidase and lipase inhibitory capabilities.

Compared to other edible flowers and legumes, *A. inaequidens* demonstrates competitive or superior enzyme inhibitory activity. For instance, α-amylase inhibition by *A. inaequidens* (EPE IC_50_ = 1.8 mg/mL) is comparable to *Passiflora tripartita* (1.72 mg/mL), *Phaseolus vulgaris* (2.69 mg/mL), and *Glycine max* (2.25 mg/mL) [54,55]. For α-glucosidase, *A. inaequidens* (DEPE IC_50_ = 1.6 mg/mL) outperformed *Hylocereus megalanthus* (8.69 mg/mL), *Dregea volubilis* (3.78 mg/mL), *Cosmos sulphureus* (5.62 mg/mL), and *Bougainvillea glabra* (5.21 mg/mL) [55,56,57]. Similarly, *A. inaequidens* also demonstrated higher lipase inhibition (DEPE IC_50_ = 1.4 mg/mL) than *C. sulphureus* (4.6 mg/mL) and *B. glabra* (5.14 mg/mL) [57]. These findings suggest that *A. inaequidens* flowers possess significant potential as natural inhibitors of key digestive enzymes, especially after simulated digestion.

## 4. Conclusions

Our findings highlight the *A. inaequidens* flower as a highly promising candidate for development as a natural functional food. Its nutritional profile marked by high fiber content and low levels of calories and fat makes it well suited for inclusion in health-conscious diets. Although the digestive process significantly reduced the levels of phenolic compounds, flavonoids, and antioxidant activity, a substantial degree of bioaccessibility was retained, particularly for flavonoids. Interestingly, digestion had a differential effect on the flower’s enzyme inhibitory properties: while its ability to inhibit amylase decreased, its inhibitory effects on glucosidase and lipase increased. These results suggest potential benefits for glycemic control and lipid metabolism. Overall, the evidence strongly supports the incorporation of *A. inaequidens* as a functional ingredient with valuable bioactive properties that may contribute to improved health outcomes.

## Figures and Tables

**Table 1:** Results of the proximate analysis of the agave flower.

Nutritional Information	Wet Basis (%)	Dry Basis (%)
Moisture	88.70 ± 12.6	-
Crude Fiber	0.85 ± 0.09	7.52 ± 0.98
Ash	0.69 ± 0.05	6.11 ± 0.52
Crude Protein	0.36 ± 0.02	3.19 ± 0.26
Crude Fat	0.22 ± 0.03	1.95 ± 0.16
Nitrogen-Free Extract	10.03 ± 1.2	81.24 ± 10.8
Energy content (Kcal/100g)	43.54 ± 2.8	

Values represent the samples mean ± SD (standard deviation, *n* = 3).

**Table 2:** Percentage yield of Agave flower extracts and fractions.

Sample	Yield
Methanolic extract (MeOH)	3.68 ± 0.2
Enriched Phenolic Extract (PE)	0.45 ± 0.08
EE fraction in ethyl acetate (EPE)	0.15 ± 0.01
Digested fraction of EPE (DEPE)	0.03 ± 0.005

Values represent the samples mean ± SD (standard deviation, *n* = 3).

**Table 3:** Total phenolic and flavonoid content and bioaccessibility percentage of phenolic compounds from undigested and digested Agave flower extracts.

Sample	Phenols [mg Eq de GA/g de Sample]	Flavonoids [mg Eq de Cat/g de Sample]
MeOH	^c^ 16.4 ± 2.0	^c^ 8.4 ± 0.8
PE	^b^ 21.8 ± 3.0	^b^ 11.2 ± 2.0
EPE	^a^ 46.1 ± 7.0	^a^ 25.3 ± 4.0
DEPE	^d^ 7.6 ± 0.5	^b^ 14.6 ±1.0
* FW	138.0 mg ± 21.0/100 g	8.0 mg ± 1.2/100 g
* DFW	21.0 mg ± 0.4/100 g	4.6 mg ± 0.3/100 g
Bioaccessibility (%)	15.22 ± 1.9	57.5 ± 2.5

Letters (a–d) represent significant differences (*p* < 0.5) between treatments. * Results expressed in fresh weight. MeOH: methanolic extract; PE: phenolic extract; EPE: enriched phenolic extract fractionated with ethyl acetate; DEPE: enriched digested phenolic extract, FW: fresh weight; DFW: digested fresh weight.

**Table 4:** Antioxidant/anti-radical activity of undigested and digested agave flower extracts by three different methods.

Sample	ABTS [μM/mgExt]	DPPH (IC_50_) [mg/mL]	AAPH (IC_50_) [μg/mL]
MeOH	^c^ 19.0 ± 3.0	^b^ 0.93 ± 0.08	-
PE	^b^ 27.0 ± 2.0	^c^ 0.61 ± 0.07	-
EPE	^a^ 42.0 ± 7.0	^d^ 0.43 ± 0.05	^b^ 70.43 ± 2.8
DEPE	^d^ 8.0 ± 0.6	^a^ 4.2 ±0.1	>100
Trolox	-	^e^ 0.012 ± 0.01	-
Ascorbic acid	-	-	^a^ 19.37 ± 0.5
* FW	5.7 μM/100 g	-	-
* DFW	1.9 μM/100 g	-	-

Letters (a–e) represent significant differences (*p* < 0.5). * Results expressed in fresh weight. Trolox: positive control (polar analog of Vitamin E). Ascorbic acid: positive control. MeOH: methanolic extract; PE: phenolic extract; EPE: phenolic extract enriched with ethyl acetate; DEPE: enriched digested phenolic extract; FW: fresh weight; DFW: digested fresh weight.

**Table 5:** Half maximal inhibitory concentration (IC_50_) of undigested and digested agave flower extracts on three digestive enzymes.

Sample	α-Amilase (IC_50_) [mg/mL]	α-Glucosidase (IC_50_) [mg/mL]	Lipase (IC_50_) [mg/mL]
MeOH	>3.0	>3.0	>3.0
PE	>3.0	^c^ 1.2 ± 0.1	>3.0
EPE	^b^ 1.8 ± 0.1	^a^ 2.7 ± 0.6	>3.0
DEPE	^a^ 2.1 ± 0.4	^b^ 1.6 ± 0.2	^a^ 1.4 ± 0.2
* Control	^c^ 0.9 ± 0.1	^d^ 0.2 ± 0.05	^b^ 0.25 ± 0.07

Letters (a–d) represent significant differences (*p* < 0.5) between treatments. * Acarbose was used as a control for α-amylase and α-glucosidase, and orlistat was used for lipase. MeOH: methanolic extract; PE: phenolic extract; EPE: enriched phenolic extract fractionated with ethyl acetate; DEPE: enriched phenolic extract digested.

## Data Availability

The original contributions generated for this study are included in the article; the data presented in this study are available on request from the corresponding author.
